# Regional Differences in Muscle and Fascial Tissue Stiffness in the Rectus Femoris Are Dependent Upon Localised Stretching

**DOI:** 10.1002/ejsc.70109

**Published:** 2026-02-25

**Authors:** Cameron D. Ley, Eduardo Martinez Valdes, Conall F. Murtagh, Jonathan Power, Barry Drust

**Affiliations:** ^1^ School of Sport, Exercise and Rehabilitation Sciences University of Birmingham Birmingham UK; ^2^ Sports Science Department Liverpool Football Club and Athletics Ground Ltd Liverpool UK; ^3^ Medical Department, Liverpool Football Club AXA Training Centre Liverpool UK

**Keywords:** biomechanics, injury & prevention, musculoskeletal, shear wave elastography

## Abstract

Mechanical properties of the deep muscle fascia are important in myofascial force transmission and injury; however, its investigation by shear wave elastography (SWE) in the literature is minimal. Regional differences in biarticular muscle stiffness have implications in mechanism of injury. To determine region‐specific differences in rectus femoris (RF) fascia and muscle stiffness, 20 healthy participants completed two visits in which RF fascia (FAS) and superficial (SUP) and deep (DEEP) muscle regions were assessed by SWE in three muscle regions (proximal ‐ PROX, medial ‐ MED and distal ‐ DIST) and at three muscle lengths (relaxed ‐ REL, neutral ‐ NEU and passively stretched ‐ PAST). DEEP was consistently stiffer than SUP muscle tissue (all *p* < 0.01) in all conditions and regions, except for REL PROX. Regional differences in SWV in all of FAS, SUP and DEEP were dependent upon local strain. Hip extension increased proximal tissue stiffness above medial and distal regions (all *p* < 0.001) and conditions of hip flexion (*p* < 0.001, *p* = 0.004 and *p* = 0.002, respectively). Similarly, knee flexion increased distal tissue stiffness above conditions of knee extension (all *p* < 0.001). Stretching the muscle by hip extension and knee flexion (PAST) removed differences between the regions in FAS, SUP and DEEP and increased medial SWV above REL and NEU (all *p* < 0.001). These results provide novel insight into regional differences in biarticular muscle and fascial tissue stiffness, implying that local strain increases stiffness in the adjacent region. These findings may have implications in force generation and region‐specific mechanisms of injury.

## Highlights

- Measured by shear wave elastography, deep rectus femoris muscle tissue is stiffer than superficial muscle tissue, regardless of measurement location.
- Regional differences in biarticular rectus femoris muscle and fascia stiffness are dependent on localised tension stress altered by manipulating joint angles. Hip extension increases proximal stiffness and knee flexion increases distal stiffness.
- Localised responsiveness to tension in biarticular muscles may have implications in region‐specific mechanisms of injury.

## 1 Introduction

The rectus femoris (RF) is a biarticular muscle involved in hip flexion and knee extension, making it important in running and kicking. The RF has the highest injury occurrence of the quadriceps muscle group (Cross et al. 2004; Mendiguchia et al. 2013). High levels of lower limb stiffness are associated with greater injury risk (Pickering Rodriguez et al. 2017; Watsford et al. 2010). These studies utilised performance tests to assess stiffness which are not muscle‐specific, instead providing insight into whole lower limb stiffness. This does not provide detailed data on stiffness of isolated muscle groups. Consequently, other approaches are needed to noninvasively estimate stiffness of specific tissues.

Shear wave elastography (SWE) is an ultrasound‐based technique developed for clinical assessment of homogenous tissue stiffness to aid the diagnosis of liver cirrhosis and breast masses (Cosgrove et al. 2012; Frulio and Trillaud 2013). It has more recently been applied to the musculoskeletal (MSK) system, where it has been validated against the gold standard tensile testing (Kodesho et al. 2021b). SWE allows reliable measurement of a specific region of interest (ROI), allowing different tissues and regions within a tissue to be assessed independently (Ley et al. 2025a). This allows specific assessment of individual muscles in isolation from surrounding structures, thereby providing insight that performance tests do not. This is particularly relevant due to the anisotropy of muscle tissue, which is not homogenous from origin to insertion.

Myofascial injury is most common in the proximal third of the RF (McAleer et al. 2022). Therefore, there is a clear need to understand differences in regional RF mechanical properties and how these change at different hip and knee angles caused by movement. Kodesho et al. (2021a) quantified regional RF muscle stiffness via SWE throughout passive knee flexion with participants supine, finding stiffness to be consistently higher proximally. Since hip angle was not adjusted, these findings may be accounted for by preferential stretching of the proximal RF at the hip. Therefore, there is a need to assess regional RF stiffness manipulating muscle length at both the hip and knee joints.

Although muscle physiology and mechanics has been extensively researched, the deep fascia has historically been overlooked in the literature. However, due to its role in force transmission and contribution to passive muscle mechanics, researchers have begun to assess its properties (Marcucci et al. 2019; Wilke et al. 2019). Kawai et al. (2021) found fascial stiffness measured by SWE to be elevated following injury compared to the uninjured contralateral limb. This highlights the importance of understanding fascial tissue stiffness in developing interventions to prevent injury and improve rehabilitation. The literature assessing fascial stiffness is sparse, with more research required to understand its mechanical properties at rest in healthy, uninjured subjects, before researchers can explore the effects of exercise and injury on fascial stiffness.

The aims of the present study were to determine (1) whether there is a difference between superficial and deep muscle tissue stiffnes, and (2) how changes in muscle length manipulated by hip and knee angle influence regional differences in RF fascial and muscle tissue stiffness.

## 2 Materials and Methods

### 2.1 Participants

Twenty participants (10 males and 10 females; age: 26.3 ± 4.0 years; height: 1.70 ± 0.10 m; weight: 67.7 ± 12.7 kg and BMI: 23.2 ± 2.8 kg/m^2^) volunteered for this study. All participants reported regularly completing at least 150 min moderate or 75 min vigorous physical activity per week as per our inclusion criteria. Participants had no history of MSK injury in the examined limb. Ethical approval for the study was granted by the Science, Technology, Engineering and Mathematics Committee (ERN_0432‐Apr2023), all procedures conformed to the Declaration of Helsinki and participants provided written informed consent. Sample size was determined via a power analysis (G* Power software) based on previous research with statistical power set at 0.80 and an alpha level of 0.05 (Taş and Salkın, 2019).

### 2.2 Experimental Design

Participants attended a familiarisation session and two experimental visits on separate days within a 7‐day period. Experimental visits were completed at the same time of day to avoid effects of routine and circadian rhythm. Participants were asked to refrain from lower body exercise and consumption of alcohol and recreational drugs 24h prior to each visit. Measurements were repeated twice per visit, with 30 min rest between measurements, wherein participants remained seated. The mean of these four measurements was used for statistical analysis.

### 2.3 Experimental Procedures

**FIGURE 1:**
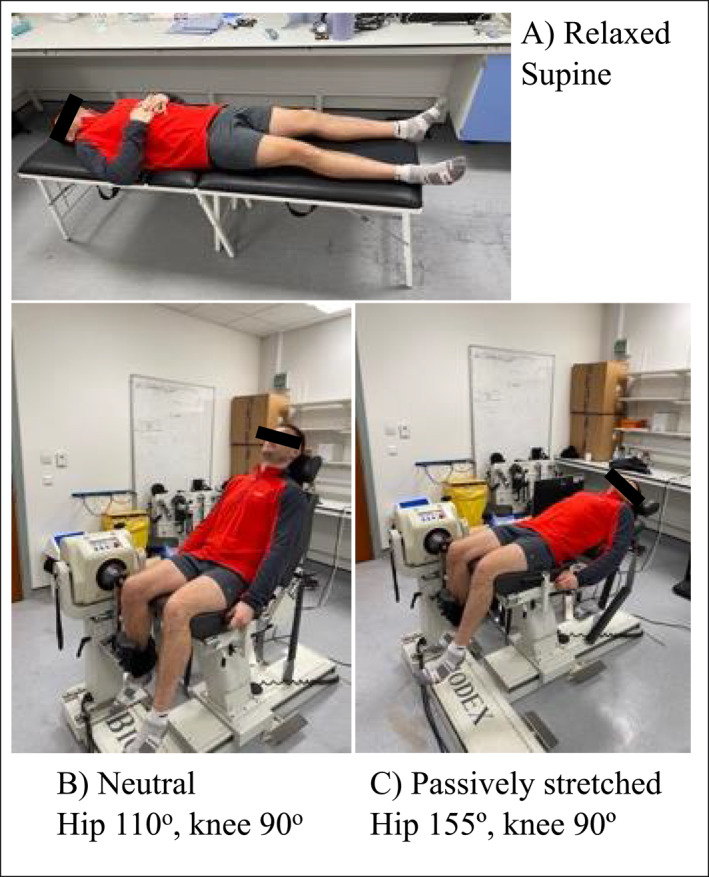
Participant positioning in the three different positions to manipulate rectus femoris muscle length (A) relaxed, (B) neutral and (C) passively stretched.

Before starting the experiment, participant height (SECA, Hamburg Germany) and weight (Ohaus ChampII, New Jersey USA) were measured, then participants rested in the laboratory, set at 21°C, for 10 min to stabilise body conditions, as per previous research (Zhou et al. 2022). Muscle stiffness was estimated at three sites along the RF muscle: proximal (PROX), medial (MED) and distal (DIST) (15%, 50% and 85% of muscle length, respectively). Muscle length was determined with participants supine using B‐mode ultrasound with a 16‐linear array probe (50 mm, 4–15 MHz) (LOGIQ S8 GE Healthcare, Milwaukee, USA), to identify the proximal and distal musculotendinous junctions. These sites were marked on the skin using a permanent marker, and muscle length (cm) measured using a measuring tape. Stiffness was estimated in three conditions to manipulate muscle length: relaxed, neutral and passively stretched. In the relaxed (REL) condition, participants were supine on an examination table. In the neutral (NEU) condition, participants sat in an isokinetic dynamometer (IKD) (Biodex System 3 Dynamometer, Biodex Medical Systems) with the hip fixed at 110° relative to full hip flexion and 90° knee flexion. In the passively stretched (PAST) condition, participants laid down in the IKD, with the hip extended at 155° relative to full hip flexion and 90° knee flexion (Figure 1). These positions were chosen to alter muscle length, with localised manipulation of muscle strain. In REL, the RF was stretched proximally; in NEU, the RF was stretched distally and in PAST, the RF was stretched both proximally and distally. To ensure consistency of measurements at the different sites between the conditions, the location of the ultrasound transducer was marked on the skin using permanent marker and anatomical landmarks were identified on the B‐mode image. During measurements, participants remained still and relaxed, avoiding muscle contractions. The conditions (REL, NEU and PAST) were chosen following pilot work to maximise participant comfort and ensure they were able to fully relax. The dominant limb, defined as participants' self‐reported leg preferred to kick a football, was selected for assessments.

### 2.4 Shear Wave Elastography

**FIGURE 2:**
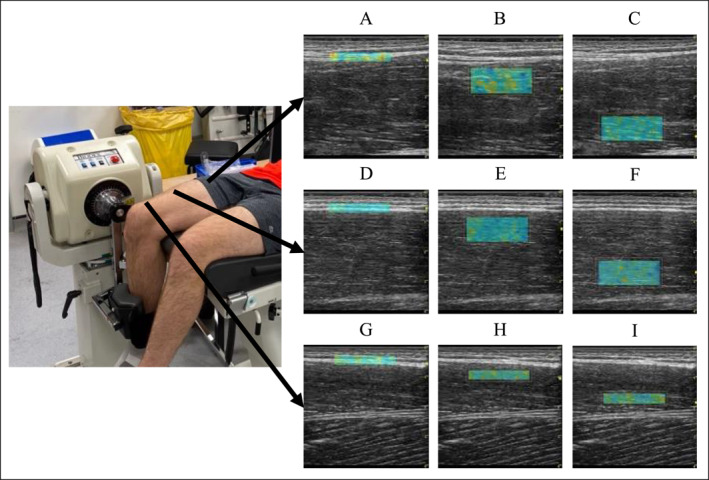
Schematic displaying the regions of interest used for shear wave velocity measurements in proximal, medial and distal locations with the participant in the passively stretched condition. Proximal (A) fascia, (B) superficial muscle, (C) deep muscle; medial (D) fascia, (E) superficial muscle, (F) deep muscle; distal (G) fascia, (H) superficial muscle and (I) deep muscle.

Stiffness was estimated via SWE using an ultrasound device equipped with SWE on a 9‐linear array probe using an MSK preset (44 mm, 2–8 MHz) (LOGIQ S8 GE Healthcare, Milwaukee, USA) which was positioned longitudinally to muscle fascicle orientation. All shear wave velocity (SWV) measurements were performed by a PhD student with 1 year of ultrasonography and MSK SWE experience (CDL). At each measurement site (PROX, MED and DIST) in all conditions (REL, NEU and PAST), SWV was estimated at three different tissue sites: fascia (FAS), superficial muscle (SUP) and deep muscle (DEEP). For measurements of FAS and distal SUP and DEEP, a 2.2cmx0.5 cm region of interest (ROI) was selected. For proximal and medial SUP and DEEP measurements, a larger ROI of 2.2cmx1.0 cm was selected (Figure 2). The thinner ROI size was used to measure DIST muscle as participants' muscle there is thinner, and this prevented overlap of ROIs between SUP and DEEP muscle measurements as shown in Figure 2. The same size was used to measure FAS as it is the minimum thickness allowed on the GE healthcare US device, therefore minimising the amount of adjacent tissue captured in the measurements. Depth of ROI positioning varied between participants based on the thickness of the muscle and its overlying subcutaneous adipose tissue and other fibrous structures up to a maximum of 4.50 cm. Subcutaneous adipose tissue thickness was measured using the measuring tool on the US device from the centre of the B‐mode image, as per Ley et al. (2025b), and is reported in Supporting Information S2. Fascia thickness was measured in the same way and is also reported in Supporting Information S2. This ROI displayed a real‐time shear wave elastogram colour map, which was recorded for 15–20s, with acquisition of new frames every 2.5s. If there were voids or saturation in the elastogram, the ROI and transducer positions were adjusted to optimise image quality. The three most visually similar consecutive frames were used for image processing, wherein mean SWV (m/s) of the ROI was measured. The mean SWV values from these three images were used for further analysis. When voids could not be avoided, frames with the least saturation were selected for analysis, this was done so by visual inspection and only images with voids covering less than 20% of the shearwave map were considered for further analysis (Dieterich et al. 2020; Ley et al. 2025a). Since we were measuring passive muscle and fascia tissue, SWV was consistently significantly below the device's upper limit, minimising presence of voids. A mechanical arm (Manfrotto, Italy) was used to ensure stability of the ultrasound transducer during measurements and avoid compression of the underlying tissue (Contreras‐Hernandez et al. 2024). Extra care was taken to avoid compression by applying a thick layer of acoustic coupling gel (> 1 mm) to create a gel standoff, which could be visualised on the B‐mode image alongside unchanged subcutaneous adipose and muscle tissue thicknesses. This protocol is reported according to guidance provided in a scoping review of MSK SWE methodology conducted by Cipriano et al. (2022).

### 2.5 Statistical Analysis

All statistical analysis was conducted using IBM SPSS software (version 29). We analysed SWV as this raw, unprocessed data does not assume the heterogeneity and isotropy of tissue that shear modulus (kPa) does, therefore providing a more accurate estimation of stiffness in the MSK context (Davis et al. 2019; Lee et al. 2016). All data were normally distributed, determined by the Shapiro–Wilk test. Intraclass correlation coefficients (ICCs) and standard error of measurements were calculated to determine inter and intraday relative and absolute reliability, respectively, of SWV measurements, as is recommended in the literature (Ley et al. 2025a). To assess differences between measurement depths (SUP and DEEP), paired *T*‐tests were conducted at each location under each condition, with Cohen's d indicating effect size. To determine the effect of the different conditions on location‐specific SWV, at each measurement depth (FAS, SUP and DEEP), a 3 location (PROX, MED and DIST) by 3 condition (REL, NEU and PAST) two‐way repeated measures ANOVA was performed, with partial eta squared (η^2^_p_) indicating effect size. If sphericity was violated, as indicated by Mauchly's test of sphericity, Greenhouse–Geisser corrections were used. Post hoc pairwise comparisons were conducted using the Bonferroni correction to investigate where the differences were significant. These analyses were conducted with sex as a between‐subject variable. Values reported in text and graphs are mean ± SD. For all analyses, statistical significance was set at *α* < 0.05.

## 3 Results

### 3.1 Reliability

Interday reliability of SWV measurements averaged an ICC of 0.804 and a standard error of measurement of 0.166 m/s. Intraday reliability of SWV measurements averaged an ICC of 0.688 and a standard error of measurement of 0.223 m/s. Full reliability results are reported in Supporting Information S1.

### 3.2 Differences in Superficial and Deep Rectus Femoris Stiffness

**FIGURE 3:**
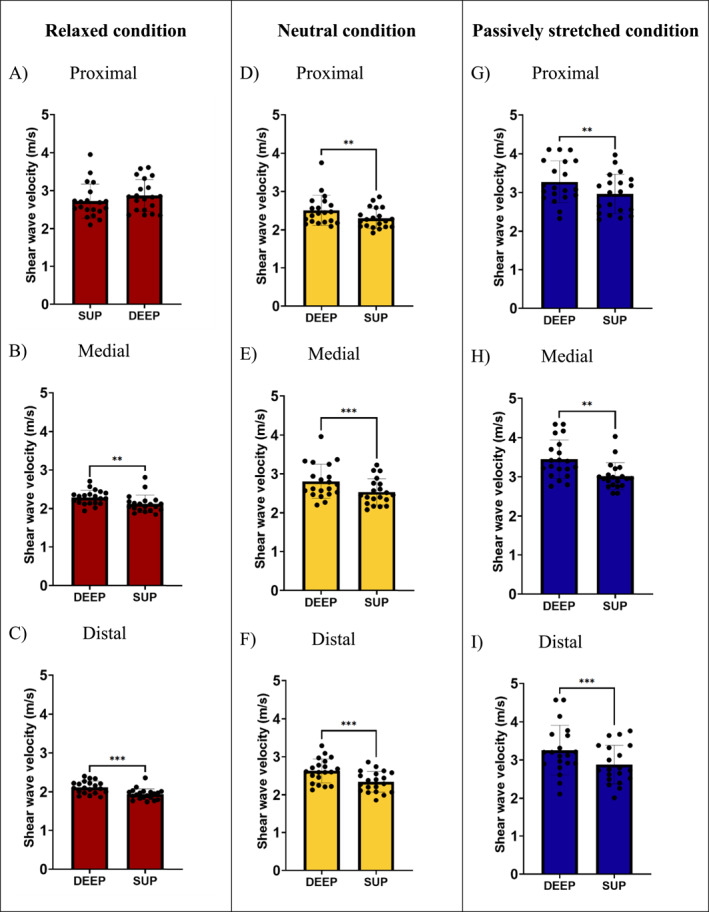
Differences in shear wave velocity between superficial and deep muscle tissue. A–C relaxed condition, in order of proximal, medial and distal regions. D–F neutral condition, in order of proximal, medial and distal regions. G–I passively stretched condition, in order of proximal, medial and distal regions. **p< 0.01 and ***p < 0.001.

Proximally in the relaxed condition, there were no significant differences between measurement depths within muscle (*p* = 0.222 and *d* = −0.282) (Figure 3A). In every other region and condition; however, deep muscle tissue SWV is consistently higher than in superficial muscle tissue (REL MED *p* = 0.002 and *d* = −0.797; REL DIST *p* < 0.001 and *d* = −0.915; NEU PROX *p* = 0.001 and *d* = −0.834; NEU MED *p* < 0.001 and *d* = −1.043; NEU DIST *p* < 0.001 and *d* = −1.228; PAST PROX *p* = 0.005 and *d* = −0.705; PAST MED *p* = 0.003 and *d* = −0.750 and PAST DIST *p* < 0.001 and *d* = −1.008) (Figure 3B–I).

### 3.3 Regional Differences

**FIGURE 4:**
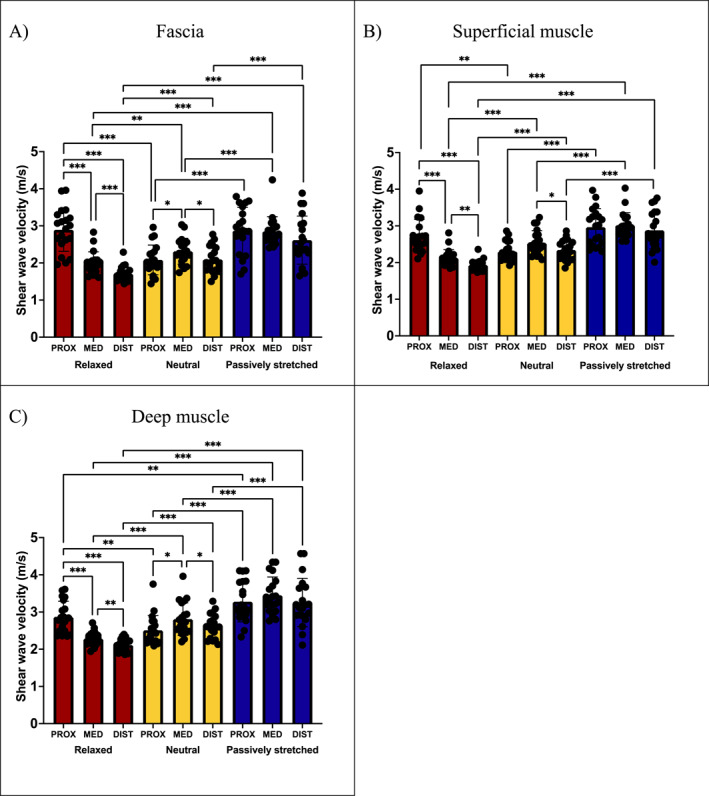
Regional differences in shear wave velocity of rectus femoris fascial and muscle tissue between conditions. REL, relaxed condition; NEU, neutral condition; PAST, passively stretched condition; PROX, proximal region; MED, medial region and DIST, distal region. Regional and conditional differences in SWV at (A) the fascia, (B) superficial muscle and (C) deep muscle. *p< 0.05 **, *p*< 0.01 and ***p< 0.001. 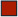 REL. 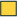 NEU. 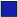 PAST.

At FAS, SUP and DEEP, there were significant main effects of location (*p* < 0.001 and $\eta_{p}^{2}$ = 0.464; *p* < 0.001 and $\eta_{p}^{2}$ = 0.341 and *p* = 0.014 and $\eta_{p}^{2}$ = 0.245, respectively), position (*p* < 0.001 and $\eta_{p}^{2}$ = 0.764; *p* < 0.001 and $\eta_{p}^{2}$ = 0.829 and *p* < 0.001 and $\eta_{p}^{2}$ = 0.788, respectively), and significant location × position interactions (*p* < 0.001 and $\eta_{p}^{2}$ = 0.646; *p* < 0.001 and $\eta_{p}^{2}$ = 0.552 and *p* < 0.001 and $\eta_{p}^{2}$ = 0.638, respectively). In all tissues (FAS, SUP and DEEP), SWV is higher proximally when the hip is extended in the relaxed (*p* < 0.001, *p* = 0.004 and *p* = 0.002, respectively) and passively stretched conditions (all *p* < 0.001) than when it is flexed in the neutral condition. Similarly, in all of FAS, SUP and DEEP, SWV is higher distally when the knee is flexed in the neutral (all *p* < 0.001) and passively stretched conditions (all *p* < 0.001) than when it is extended in the relaxed condition. This is the same for medial tissue, wherein FAS, SUP and DEEP SWVs are higher with knee flexion in the neutral (*p* = 0.001, *p* < 0.001 and *p* < 0.001, respectively) and passively stretched conditions (all *p* < 0.001) than in the relaxed condition. When the hip and knee are extended in the relaxed condition, SWV is higher for all of FAS, SUP and DEEP proximally than medially (all *p* < 0.001) or distally (all *p* < 0.001) and higher medially than distally (*p* < 0.001, *p* = 0.001 and *p* = 0.004, respectively). However, when the knee is flexed in the passively stretched condition, these regional differences disappear due to the bidirectional tension. Furthermore, when the hip is flexed in the neutral condition, SWV proximally is no longer higher than it is distally (Figure 4). Finally, in the passively stretched condition, when the muscle is under the highest level of tension, SWV of FAS, SUP and DEEP are higher medially than in the relaxed (all *p* < 0.001) or neutral conditions (all *p* < 0.001).

## 4 Sex Differences

In FAS, there is a significant location × sex interaction (*p* = 0.034 and $\eta_{p}^{2}$ = 0.171), with post hoc analysis revealing that, distally, males have significantly greater SWV than females (*p* = 0.002). In SUP, there is a significant location x position × sex interaction (*p* = 0.003 and $\eta_{p}^{2}$ = 0.197), with post hoc analysis revealing that, distally, males have significantly greater SWV than females in NEU (*p* = 0.025) and PAST (*p* = 0.012). In DEEP, there is a significant location x position × sex interaction (*p* = 0.022, $\eta_{p}^{2}$ = 0.154), with post hoc analysis revealing that, in PAST, males have significantly greater SWV than females in medial (*p* = 0.023) and distal (*p* = 0.015) tissue.

## 5 Discussion

Results from the present study indicate that (1) deep RF muscle is stiffer than superficial muscle and (2) fascial and muscle tissue SWV is higher proximally when the hip is extended, higher distally when the knee is flexed, and highest when both the hip is extended, and knee is flexed. In addition, this study highlights the susceptibility of the biarticular RF to differential strain based on altering hip and knee angles, which may have implications for force generation, and injury mechanisms and prevention. It also supports the existing literature by finding greater levels of muscle stiffness in males than in females.

### 5.1 Muscle Depth

To our knowledge, this is the first study to compare measurement depths of RF muscle stiffness. In all conditions and regions, except for proximally in the relaxed condition, deep muscle exhibited a higher SWV than superficial muscle. These findings are concordant with findings in other muscles, including the supraspinatus, multifidus and forearm muscles (Jiang et al. 2023; Murillo et al. 2019; Wang et al., 2020). These findings may be due to deeper muscle fibres attaching to aponeuroses. These thick fibrous layers of connective tissue exert an anchoring effect on deeper muscle fascicles, increasing fascicle strain (Marcucci and Reggiani 2020). This is supported by findings in the gastrocnemius muscle, where a stiffer aponeurosis was associated with stiffer adjacent muscle tissue (Slane et al. 2017). This would explain our findings, wherein deeper muscle tissue closer to the RF aponeurosis was stiffer than more superficial muscle tissue as a function of tension from the connecting deep aponeurosis. However, it must be considered that these results may be due to a limitation with SWE. Greater acquisition/ROI depths yield higher SWV values in muscle and gel phantom models (Shin et al. 2016; Wang et al. 2014; Wang et al. 2020). This is likely due to a decline in intensity of the acoustic radiation force impulse as it transmits to deeper tissue. This phenomenon is also the cause of the attenuation effect, wherein there are greater void areas and areas of excessively high and low SWVs reported within deeper elastograms. Accordingly, it has been recommended that a SWV acquisition depth of < 3 cm is most viable for assessing muscle tissue (Wang et al. 2020). Further methodological research is required to determine the viability of SWE as a method of assessing deep tissue stiffness.

With growing interest in the fasciae, our findings may have implications regarding force transmission and injury risk in the RF fascia. More research is needed to determine normative regional stiffness of overlying fasciae in other muscles so that practitioners can more confidently interpret SWV data (Barzegar et al. 2023; Wilke et al. 2022). This is particularly relevant when attempting to gain insight into injury risk, rehabilitation progress and readiness to play because stiffness of injured fascia has been shown to remain elevated years after injury occurrence (Kawai et al. 2021). Further research is required to determine best practice for assessing SWV of the deep fascia.

### 5.2 Regional Differences

To our knowledge, this is the first study to manipulate RF length at both the hip and knee and investigate stiffness of fascial and muscle tissue at different depths. Results show that higher levels of proximal fascial and muscle tissue stiffness are a result of localised tension stress caused by hip extension, since, when the hip is flexed, in NEU, there is no difference between proximal and distal regions. This regional behaviour appears characteristic of the biarticular RF muscle as the same principle is observed in the distal region. Here, fascial and muscle tissue stiffness are greater when the knee is flexed in NEU and PAST than it is in REL. There were no regional differences when the muscle was under tension stress from both tendons in PAST, evenly distributing strain along the muscle. Combined, these findings indicate that regional differences in RF stiffness are dependent upon localised tension stress applied to the tissue. These localised impacts of movement on RF stiffness may influence risk and mechanism of injury during exercise, whereby hip extension is more likely to cause muscle strain injuries proximally.

We found a higher SWV in medial RF fascial and muscle tissue in PAST than REL or NEU. This supports the well‐established research showing that increasing muscle length augments muscle stiffness (Itsuda et al. 2024; Le Sant et al. 2015). Specifically in the RF, increasing knee flexion is accompanied by increases in RF muscle stiffness with the hip flexed or extended (Coombes et al. 2018; Xu et al. 2018). Furthermore, research in the biarticular gastrocnemius medialis (GM) muscle found that both knee extension and dorsiflexion increased shear modulus (Zhou et al. 2020). This study is the first to show that this trend is the same in the deep fascia as it is in muscle.

Our results show that the RF displays a higher SWV proximally with hip extension, and higher SWV distally with knee flexion. These findings are supported by the literature, since when the knee is extended, GM stiffness is higher proximally than medially and distally (Liu et al. 2020; Zhou et al. 2019). Similarly, higher levels of strain have been observed proximally in biceps femoris (BF) cadavers with increasing anterior pelvic tilt (Mendiguchia et al. 2024). Therefore, these findings appear to be characteristic of biarticular muscles, such that stress‐induced increases in stiffness are not uniformly distributed along the muscle's length, but exacerbated in the region closest to the joint stretching the muscle.

With the known heterogeneity of muscle tissue, it is unsurprising that regional differences in mechanical properties exist within the RF. Studies utilising electromyography have shown preferential activation of proximal RF regions during hip flexion, highlighting the ability of RF motor units to work independently to fulfil the muscle's dual functions (Watanabe et al. 2021). Furthermore, Kodesho et al. (2021a) found that, with the hip extended, RF stiffness is consistently higher proximally than distally, regardless of knee joint angle. Our findings in PAST contrast these results, as we observed no significant differences between regions. This is likely due to heterogeneity in scanning position between our studies. In PAST, our participants had a hip angle of 155° to avoid hip overextension, whereas participants were supine with 180° hip angle in the study by Kodesho et al. (2021a), so participants experienced greater hip extension and more tension stress on the muscle from the proximal tendon. Moreover, our study included female participants, who likely had a higher hip ROM and greater flexibility, reducing relative tension from the proximal tendon compared with the male participants recruited by Kodesho et al. (2021a) (Nagai et al. 2021).

It is noteworthy that these trends were consistent between fascial and muscle tissue, but perhaps unsurprising, since mechanical properties are consistent between materials, wherein application of a tension stress leads to an increase in stiffness. Our results show that regional differences in RF muscle stiffness are not consistent and that regional differences in SWV of biarticular muscles must be interpreted in the context of participant joint positioning.

These findings may have implications in the regional occurrence and mechanism of RF injuries in specific phases of human movement and sporting actions, whereby rapid contractions involving knee flexion may be more likely to induce RF injury distally, and hip extension to do so proximally. However, more research is required to understand the link between regional differences in stiffness and injury location to this level of specificity.

Our findings underscore the importance of standardising SWE measurement protocols for use in research, clinical settings and in athlete screening to allow comparison of results (Cipriano et al. 2022). Changing muscle length and, in the case of biarticular muscles, its manipulation at both joints alters muscle stiffness regionally. This must be considered when clinicians and practitioners choose patient and athlete positioning when taking SWV measurements.

### 5.3 Sex Differences

Our results indicate that fascial tissue is stiffer distally in males than females; that muscle tissue stiffness is higher distally in males than females when the knee is flexed in NEU, and higher both medially and distally in males than females in PAST. These findings align with the literature, which has established that males exhibit significantly higher passive and active lower limb muscle (RF, BF, GM and tibialis anterior) stiffness than females (Lee et al. 2021; McPherson et al. 2020). Perhaps, with a higher sample size, we may have found further differences between the sexes, as Lee et al. (2021) did with double our sample. However, it is worth considering that, since our differences are localised to the region closest to the flexed knee joint, these differences may be a result of the probable lower range of motion in the male participants and thus a consequence of being at a greater relative knee joint range of motion in these conditions.

## 6 Limitations

Participants were asked to remain still and relaxed during measurements; however, we did not utilise electromyography to confirm the absence of muscle activity. Presence of muscle activity would lead to an overestimation of muscle stiffness. A scoping review by Cipriano et al. (2022) found that only 11% of SWE studies utilise EMG to confirm the absence of muscle activity. Other studies report sufficient SWE reliability without the use of EMG but future studies should compare the measurement of muscle stiffness with and without the use of EMG to determine the validity of visually monitoring participants (Taş et al. 2017). The selection of frames, instead of measuring every frame within the trial, was done so to avoid inclusion of frames with saturation and voids but may have introduced subjectivity and selection bias which was minimised through analysis being completed by the same researcher. The minimum size of a ROI on the GE healthcare ultrasound device is 2.2 cm x 0.5 cm, this meant that some adjacent nontarget tissue was included in the measurement of FAS and SWV values are not representative of solely fascial tissue. Since adjacent subcutaneous adipose and muscle tissue is less stiff than FAS, this may have led to an underestimation of FAS SWV. Future studies should therefore manually select ROIs including only the target tissue to more accurately estimate their stiffness. To enhance this, all medical technology companies should allow for smaller ROIs to be measured with US SWE. Furthermore, the GE healthcare device has a limited frame rate, a common problem with SWE, and such devices should be developed with more sophisticated systems to obtain a greater sampling frequency. Finally, we did not account for menstrual cycle phase in our 10 female participants. This was because there is very little evidence for fluctuations in muscle stiffness across the menstrual cycle or for sex hormone‐induced alterations in muscle stiffness (Saeki et al. 2019). However, this is an underinvestigated topic, with some evidence suggesting that muscle stiffness may be higher during the follicular phase than during ovulation (Khowailed et al. 2022). Although our participants all reported meeting UK government guidelines on physical activity levels, it is likely that they participate in sport to varying degrees, thus introducing heterogeneity of tissue properties between participants. Furthermore, our study only investigated RF properties in participants' dominant limb, limiting the generalisability of our findings.

## 7 Conclusions

Superficial muscle tissue is consistently less stiff than deep muscle tissue. Regional differences in RF muscle and fascia stiffness are not consistent but instead altered by manipulating regional muscle strain in response to localised tension stress. Hip extension increases proximal stiffness, and knee flexion increases distal stiffness. This localised responsiveness to tension may have implications in the region‐specific mechanisms of injury, that is, excessive hip extension is more likely to cause proximal, rather than distal, RF muscle injury.

## Funding

The authors have nothing to report.

## Conflicts of Interest

The authors declare no conflicts of interest.

## Supporting information


Supporting Information S1

Supporting Information S2
