# Supplementary material for: Regional Differences in Muscle and Fascial Tissue Stiffness in the Rectus Femoris Are Dependent Upon Localised Stretching
Source: Eur J Sport Sci. 2026 Feb 25;26(3):e70109. doi: 10.1002/ejsc.70109 (PMC12935568; doi:10.1002/ejsc.70109)
Supplement: Supplementary file 1 — Supporting Information S1 [file EJSC-26-e70109-s001.docx]

| Condition | Region | Depth | ICC (single measures) | Standard error of measurement (m/s) |
| --- | --- | --- | --- | --- |
| Relaxed | Proximal | Skin | 0.900 | 0.141 |
|  |  | Fascia | 0.883 | 0.184 |
|  |  | Superficial muscle | 0.743 | 0.219 |
|  |  | Deep muscle | 0.740 | 0.232 |
|  | Medial | Skin | 0.829 | 0.158 |
|  |  | Fascia | 0.854 | 0.122 |
|  |  | Superficial muscle | 0.711 | 0.138 |
|  |  | Deep muscle | 0.538 | 0.145 |
|  | Distal | Skin | 0.686 | 0.138 |
|  |  | Fascia | 0.743 | 0.110 |
|  |  | Superficial muscle | 0.886 | 0.055 |
|  |  | Deep muscle | 0.474 | 0.134 |
| Neutral | Proximal | Skin | 0.788 | 0.100 |
|  |  | Fascia | 0.934 | 0.110 |
|  |  | Superficial muscle | 0.653 | 0.170 |
|  |  | Deep muscle | 0.904 | 0.130 |
|  | Medial | Skin | 0.882 | 0.122 |
|  |  | Fascia | 0.686 | 0.214 |
|  |  | Superficial muscle | 0.838 | 0.145 |
|  |  | Deep muscle | 0.864 | 0.170 |
|  | Distal | Skin | 0.907 | 0.161 |
|  |  | Fascia | 0.894 | 0.122 |
|  |  | Superficial muscle | 0.662 | 0.179 |
|  |  | Deep muscle | 0.762 | 0.164 |
| Passively stretched | Proximal | Skin | 0.834 | 0.155 |
|  |  | Fascia | 0.837 | 0.270 |
|  |  | Superficial muscle | 0.907 | 0.155 |
|  |  | Deep muscle | 0.872 | 0.200 |
|  | Medial | Skin | 0.844 | 0.190 |
|  |  | Fascia | 0.912 | 0.122 |
|  |  | Superficial muscle | 0.733 | 0.197 |
|  |  | Deep muscle | 0.854 | 0.200 |
|  | Distal | Skin | 0.748 | 0.263 |
|  |  | Fascia | 0.864 | 0.253 |
|  |  | Superficial muscle | 0.815 | 0.235 |
|  |  | Deep muscle | 0.945 | 0.158 |

**Table S1**: Inter-day reliability results for shear wave elastography in relaxed, neutral and passively stretched conditions; proximal, medial and distal, regions; and skin, fascia, superficial muscle and deep muscle. Measures of relative and absolute repeatability are presented as intraclass correlation coefficient (ICC) and standard error of the measurement, respectively.

| Condition | Region | Depth | ICC (single measures) | Standard error of measurement (m/s) |
| --- | --- | --- | --- | --- |
| Relaxed | Proximal | Skin | 0.789 | 0.257 |
|  |  | Fascia | 0.887 | 0.210 |
|  |  | Superficial muscle | 0.749 | 0.257 |
|  |  | Deep muscle | 0.619 | 0.308 |
|  | Medial | Skin | 0.690 | 0.228 |
|  |  | Fascia | 0.824 | 0.138 |
|  |  | Superficial muscle | 0.596 | 0.173 |
|  |  | Deep muscle | 0.331 | 0.207 |
|  | Distal | Skin | 0.407 | 0.228 |
|  |  | Fascia | 0.652 | 0.134 |
|  |  | Superficial muscle | 0.490 | 0.118 |
|  |  | Deep muscle | 0.270 | 0.200 |
| Neutral | Proximal | Skin | 0.420 | 0.187 |
|  |  | Fascia | 0.810 | 0.182 |
|  |  | Superficial muscle | 0.546 | 0.219 |
|  |  | Deep muscle | 0.716 | 0.219 |
|  | Medial | Skin | 0.738 | 0.200 |
|  |  | Fascia | 0.691 | 0.230 |
|  |  | Superficial muscle | 0.635 | 0.232 |
|  |  | Deep muscle | 0.752 | 0.239 |
|  | Distal | Skin | 0.796 | 0.266 |
|  |  | Fascia | 0.738 | 0.197 |
|  |  | Superficial muscle | 0.676 | 0.187 |
|  |  | Deep muscle | 0.557 | 0.253 |
| Passively stretched | Proximal | Skin | 0.667 | 0.241 |
|  |  | Fascia | 0.855 | 0.255 |
|  |  | Superficial muscle | 0.808 | 0.239 |
|  |  | Deep muscle | 0.771 | 0.274 |
|  | Medial | Skin | 0.756 | 0.249 |
|  |  | Fascia | 0.808 | 0.179 |
|  |  | Superficial muscle | 0.746 | 0.200 |
|  |  | Deep muscle | 0.789 | 0.237 |
|  | Distal | Skin | 0.681 | 0.316 |
|  |  | Fascia | 0.862 | 0.259 |
|  |  | Superficial muscle | 0.826 | 0.230 |
|  |  | Deep muscle | 0.818 | 0.292 |

**Table S2**: Intra-day reliability results for shear wave elastography in relaxed, neutral and passively stretched conditions; proximal, medial and distal, regions; and skin, fascia, superficial muscle and deep muscle. Measures of relative and absolute repeatability are presented as intraclass correlation coefficient (ICC) and standard error of the measurement, respectively.
