# Supplementary material for: Regional Differences in Muscle and Fascial Tissue Stiffness in the Rectus Femoris Are Dependent Upon Localised Stretching
Source: Eur J Sport Sci. 2026 Feb 25;26(3):e70109. doi: 10.1002/ejsc.70109 (PMC12935568; doi:10.1002/ejsc.70109)
Supplement: Supplementary file 2 — Supporting Information S2 [file EJSC-26-e70109-s002.docx]

| Condition | Region | Subcutaneous adipose tissue thickness (cm) |
| --- | --- | --- |
| Relaxed | Proximal | 0.86 ± 0.52 |
|  | Medial | 0.89 ± 0.49 |
|  | Distal | 0.83 ± 0.49 |
| Neutral | Proximal | 0.97 ± 0.60 |
|  | Medial | 0.87 ± 0.49 |
|  | Distal | 0.75 ± 0.44 |
| Passively stretched | Proximal | 0.87 ± 0.55 |
|  | Medial | 0.84 ± 0.47 |
|  | Distal | 0.76 ± 0.45 |

**Table S3:** Subcutaneous adipose tissue thickness (mean ± standard deviation) in the three conditions and regions.

| Condition | Region | Fascia thickness (cm) |
| --- | --- | --- |
| Relaxed | Proximal | 0.17 ± 0.04 |
|  | Medial | 0.17 ± 0.04 |
|  | Distal | 0.17 ± 0.04 |
| Neutral | Proximal | 0.18 ± 0.06 |
|  | Medial | 0.17 ± 0.05 |
|  | Distal | 0.17 ± 0.04 |
| Passively stretched | Proximal | 0.17 ± 0.04 |
|  | Medial | 0.17 ± 0.04 |
|  | Distal | 0.17 ± 0.04 |

**Table S4:** Fascia thickness (mean ± standard deviation) in the three conditions and regions.
